# Statistical characterization of the growth and spatial scales of the substorm onset arc

**DOI:** 10.1002/2015JA021470

**Published:** 2015-10-20

**Authors:** N. M. E. Kalmoni, I. J. Rae, C. E. J. Watt, K. R. Murphy, C. Forsyth, C. J. Owen

**Affiliations:** ^1^ Mullard Space Science LaboratoryUniversity College LondonDorkingUK; ^2^ Department of MeteorologyUniversity of ReadingReadingUK; ^3^ NASA Goddard Space Flight CenterGreenbeltMarylandUSA

**Keywords:** ULF waves, THEMIS, aurora, instabilities

## Abstract

We present the first multievent study of the spatial and temporal structuring of the aurora to provide statistical evidence of the near‐Earth plasma instability which causes the substorm onset arc. Using data from ground‐based auroral imagers, we study repeatable signatures of along‐arc auroral beads, which are thought to represent the ionospheric projection of magnetospheric instability in the near‐Earth plasma sheet. We show that the growth and spatial scales of these wave‐like fluctuations are similar across multiple events, indicating that each sudden auroral brightening has a common explanation. We find statistically that growth rates for auroral beads peak at low wave number with the most unstable spatial scales mapping to an azimuthal wavelength *λ*≈ 1700–2500 km in the equatorial magnetosphere at around 9–12 *R*_*E*_. We compare growth rates and spatial scales with a range of theoretical predictions of magnetotail instabilities, including the Cross‐Field Current Instability and the Shear Flow Ballooning Instability. We conclude that, although the Cross‐Field Current instability can generate similar magnitude of growth rates, the range of unstable wave numbers indicates that the Shear Flow Ballooning Instability is the most likely explanation for our observations.

## Key Points

- Auroral beads are a repeatable signature along the substorm onset arc
- Bead brightness grows exponentially across a wide range of spatial scales
- Growth rates peak at those spatial scales predicted by shear flow ballooning

## 1 Introduction

The causal sequence of events leading to energy release and auroral breakup during substorms remains unknown, primarily due to a lack of spatial and temporal resolution when investigating the physical processes occurring within the first 2 min of substorm onset in such a vast 3‐D volume of space. The discrepancy and uncertainty in timings between magnetospheric processes and auroral signatures prior to the expansion phase have caused a controversial and currently unresolved debate over the physical process leading to the substorm expansion phase onset. This debate has predominantly focused on two substorm onset paradigms: (1) magnetic reconnection at the Near‐Earth Neutral Line (NENL) [*Baker et al.*, 1996; *Hones*, 1976] causing earthward plasma flows which destabilize the central plasma sheet or (2) a near‐Earth magnetospheric disturbance triggering current disruption (CD) in the central plasma sheet [*Roux et al.*, 1991; *Lui et al.*, 1991]. Other models include the boundary layer dynamics model [*Rostoker and Eastman*, 1987], near‐Earth geophysical onset model [*Maynard et al.*, 1996], and global Alfvénic interaction model [*Song and Lysak*, 2001]. The NENL and CD models have been most extensively discussed in the field, e.g., *Angelopoulos et al.* [2008, 2009] and *Lui*, [2009]; however, no consensus has yet been reached. Further complexity to the NENL model has since been added, e.g., *Nishimura et al.* [2010] and *Sergeev et al.* [2012], where the impacts of flow bursts on auroral breakup are discussed.

Substorm onset is marked in the ionosphere by a sudden brightening of the most equatorward auroral arc or, in some instances, the formation of a new arc that brightens [*Akasofu*, 1977] and is followed by auroral breakup. Early observations of substorm aurora provided by the Viking mission enabled the discovery of small‐scale azimuthal auroral fluctuations, nicknamed “auroral beads” [*Henderson*, 1994] or subsequently azimuthal auroral forms [after *Elphinstone et al.*, 1995] which form along the onset arc in the minutes leading up to auroral breakup. Auroral beads observed with space‐based imagery have only been sporadically reported since *Henderson* [2009].

The aim of the Time History of Events and Macroscale Interactions during Substorms (THEMIS) [*Angelopoulos*, 2008; *Sibeck and Angelopoulos*, 2008] mission is to uncover the temporal sequence of processes linked with substorms. The increased spatial coverage provided by THEMIS all‐sky imagers (ASI) [*Mende et al.*, 2008], together with its high spatial and temporal resolution, has led to the renewed interest in small‐scale azimuthal auroral beads forming along the onset arc [*Friedrich et al.*, 2001; *Liang et al.*, 2008; *Sakaguchi et al.*, 2009; *Rae et al.*, 2009a, 2010]. From here on we will refer to this phenomenon as auroral beads. Auroral beads have been interpreted in a variety of ways. *Rae et al.* [2010] and *Motoba et al.* [2012] conclude that they are the ionospheric signatures of a magnetospheric instability. In contrast, *Haerendel* [2010, 2015] interpret the origin of auroral beads as the *point of preferred entry of magnetic flux from the central current sheet of the tail* due to a current sheet collapse. The latter concludes that flow bursts are stalled due to a stop layer of the width of an ion inertial length, leading to the formation of closely spaced field‐aligned currents which are responsible for the periodic auroral beads.

*Motoba et al.* [2012] observed magnetically conjugate auroral beads in ASI data from both Northern and Southern Hemispheres and suggested that the beads have a common driver originating in the magnetosphere. In addition to these wave‐like signatures in the aurora, simultaneous magnetic pulsations of ULF waves have also been observed in the minutes surrounding substorm onset [*Mann et al.*, 2008; *Milling et al.*, 2008; *Murphy et al.*, 2009a, 2009b; *Rae et al.*, 2009a, 2009b; *Walsh et al.*, 2010; *Rae et al.*, 2011]. Moreover, these ULF pulsations are repeatably observed at frequencies similar to those observed in the auroral beads [*Rae et al.*, 2012], suggesting an inextricable link between the auroral and magnetic waves.

The previously discussed studies of auroral beads were limited to descriptions of the initial azimuthal wavelength and its temporal evolution. *Rae et al.* [2010] provide optical analysis of substorm auroral arc azimuthal wave number spectra during a single event, which demonstrates that the beading of the substorm onset arc is characteristic of an instability in the near‐Earth magnetosphere. *Rae et al.* [2010] report that the frequency, spatial scales, and growth rates of the auroral structures are most consistent with either a Cross‐Field Current Instability (where growth rates peak at ∼0.4 s^−1^) [*Lui et al.*, 1991; *Lui*, 2004] or a Shear Flow Ballooning Instability (where growth rates peak at ∼0.2 s^−1^) [*Voronkov et al.*, 1997]. However, *Rae et al.* [2010] could not identify which of these two instabilities acted during this event, nor could they definitively rule out the Kelvin‐Helmholtz, e.g., *Yoon et al.* [1996], or entropy antidiffusion instability, e.g., *Lee et al.* [1998], due to unknown magnetotail conditions.

In this paper we perform a more quantitative optical analysis to that first outlined in *Rae et al.* [2010] over multiple events that display wave‐like auroral beads along the substorm onset arc in the minutes leading to substorm onset. For each substorm and pseudo‐breakup (a sudden auroral brightening in the midnight sector, which does not lead to poleward motion or auroral breakup) event, we characterize the spatial and temporal scales of auroral bead growth and azimuthal propagation. This allows the statistical relationship between wave number and growth rate of auroral beads to be found, which we then compare with theoretical predictions of instability characteristics.

## 2 Optical Analysis

In this study, we use data from the NASA THEMIS mission ASIs. The fields of view of the ASIs form an overlapping array spanning the auroral oval across Canada and Alaska, which covers up to 12 h of local time. The THEMIS ASIs are white light auroral imagers that primarily respond to 557.7 nm (green emission) aurora [*Mende et al.*, 2008] and so throughout this study, we assume an emission altitude of 110 km. At zenith the THEMIS ASIs provide up to 1 km spatial resolution and capture images at a 3 s cadence.

**Figure 1:**
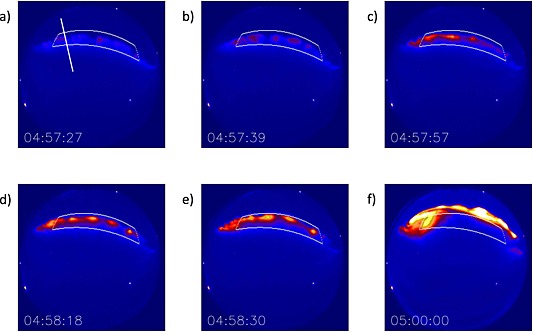
Auroral beads along the onset arc during the auroral substorm observed at GILL ASI on 2 October 2011. Lines of geomagnetic latitude at 67.8° and 68.4° and geomagnetic longitude at −33.0° and −24.0° define the field of view of our analysis and show that the onset arc is aligned with constant geomagnetic latitude. We track the temporal and spatial evolution of the auroral beads within this white box in our subsequent analysis. The line perpendicular to the arc along which we use for the keogram in Figure 2a is shown in Figure 1a. The formation and evolution of the beads are observed with time. After 04:58:30 UT (Figure 1e) the aurora expands poleward out the box, as can be seen at a later time in (Figure 1f).

An example of a typical isolated substorm onset event used in this study occurs at 04:57 UT on 2 October 2011 and is presented in Figure 1. This event is characterized by a sudden brightening of the auroral arc at  04:57:30 UT followed by poleward expansion. Figures 1a–1f show the raw data from the ASI at Gillam (GILL) and the formation and evolution of auroral beads during the 2 October 2011 event. The white box in Figure 1 shows the portion of the ASI field of view used in subsequent analysis. Figure 1a shows the initial formation of bead‐like azimuthal structure along the most equatorward auroral arc. Subsequently, the beads brighten and are visible at regular intervals along the auroral arc (Figures 1b–1d. In Figure 1e, the arc brightens further and starts to move poleward and finally the arc shows nonregular structuring (or “breaks up”) and expands poleward out of the field of view of the analysis box. We limit our analysis to the time interval before the aurora expands outside of the white box.

**Figure 2:**
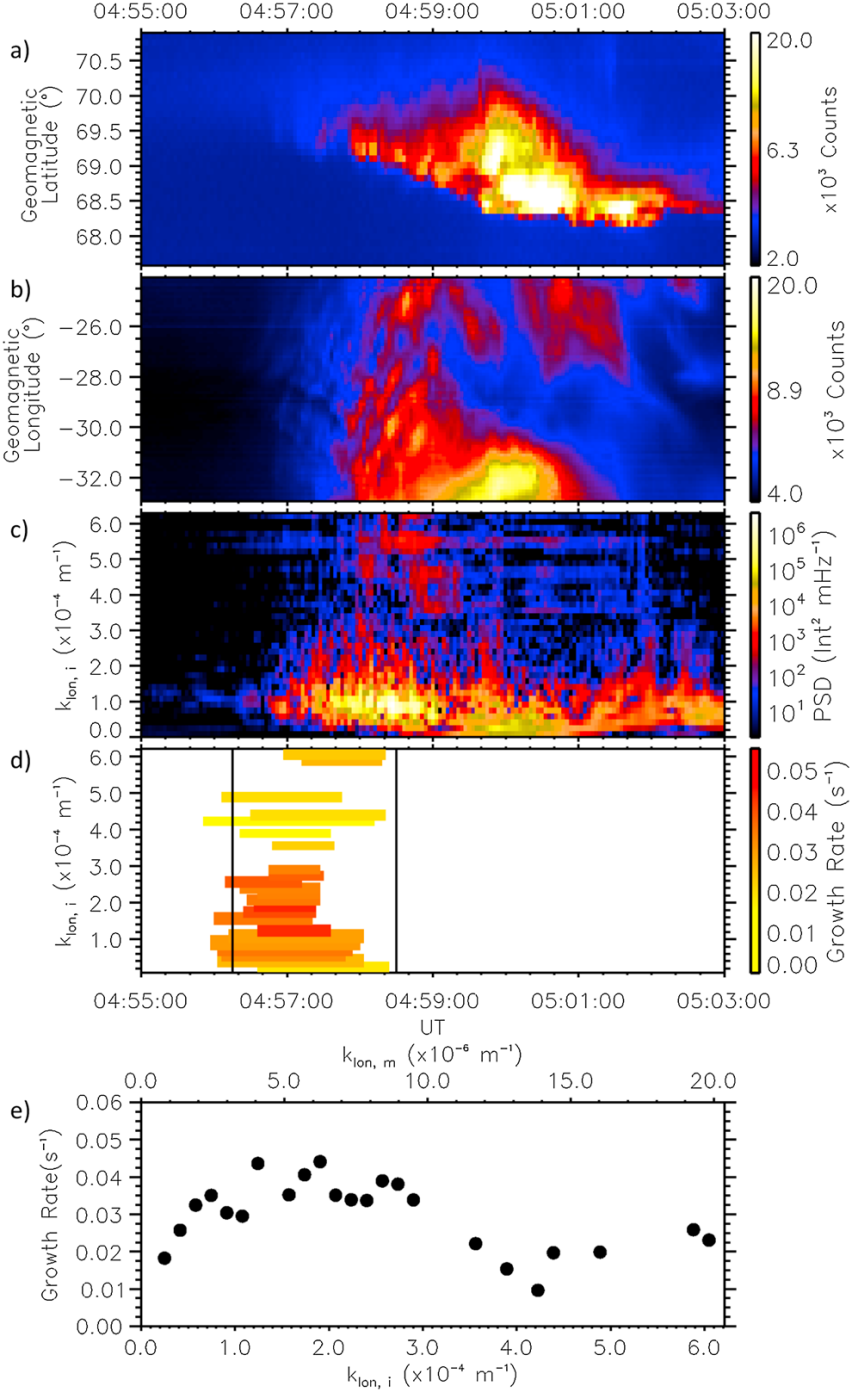
Optical analysis for substorm at Gillam on 2 October 2011. (a) North‐south keogram to show auroral brightening and poleward propagation. (b) East‐west keogram along a line of geomagnetic latitude (as a function of longitude) to track periodic azimuthal structure along the onset arc. (c) Power Spectral Density as a function of longitudinal wave number measured in the ionosphere, *k*_lon,*i*_. (d) Periods of exponential growth for each *k*_lon,*i*_, where the duration of exponential growth is marked by the length of the horizontal line and the growth rate denoted by the color. The interval encompassing substorm onset is marked by the vertical lines. Only wave numbers that grow for over 30 s and start within 1 standard deviation of the median start time are used and (e) growth rate as a function of azimuthal wave number for those wave numbers that demonstrate exponential growth according to (Figure 2d).

Figure 2a shows a north‐south slice (keogram) perpendicular to the arc orientation, which is aligned geomagnetically east‐west. The line along which the keogram is made is shown in white in Figure 1a.

**Figure 3:**
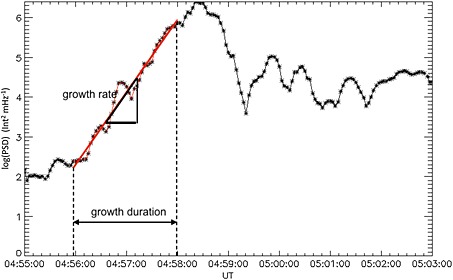
Exponential growth rate determination. The log of the power from the power spectral density (Figure 2c) for a single wave number, *k*_lon_=0.9 × 10^−4^ m^−1^, plotted against time shows the times between which there is exponential growth denoted by the linear fit (red). The growth rate is given by the gradient of the fit.

In general, the substorm onset arc is closely aligned with geomagnetic latitude [*Akasofu*, 1964], a fact we utilize in order to characterize the spatial and temporal behavior of the auroral bead evolution through substorm onset within our denoted field‐of‐view. Figures 2b–2e demonstrate our analysis as performed on the 2 October 2011 substorm observed at GILL. Figure 2b shows auroral intensity within our box as a function of geomagnetic longitude (east‐west keogram) along the onset arc. The clear formation of auroral beads (Figure 2b) along the substorm onset arc are first observed at the same time as the rapid auroral brightening (∼04:57:30 UT). The periodic auroral beads initially have a westward phase propagation but interestingly develop eastward phase propagation around 20 s later. Figure 2c shows the time evolution of the spatial Fourier transform in the longitudinal direction in order to quantify the spatial periodicity of the auroral beads during this substorm. In order to reduce edge effects, we detrend the data in time and space using a 2‐D Hanning window and reapply the appropriate corrective factor to recover the correct power spectral density (PSD) values. The dynamic PSD in Figure 2c shows that the highest powers are located at *k*_lon_≈0.5–1.5 × 10^−4^ m^−1^ during the initial beading. It is important to note that the power over a range of *k*_lon_ grows exponentially over an interval that encompasses the visually identified onset at 04:57:30 UT. Hence, for each *k*_lon_, we identify intervals of exponential growth that occur during substorm onset. Figure 3 shows an example of an exponentially growing mode during this event at *k*_lon_=0.9 × 10^−4^ m^−1^. We use an algorithm to detect exponential growth of the power spectral density time series. We use a linear fitting method based upon the least absolute deviations technique to determine growth rate, duration, and start and end time (given by the start and end of the linear fit) for each *k*_lon_. This algorithm requires (a) that exponential growth must be continually present over a duration longer than 30 s, since this is the typical period of a bead fluctuation [*Rae et al.*, 2010], (b) that it occurs before the aurora expand poleward out of the analysis field of view, and (c) that it must start within the window identified to contain substorm onset. In order to define a reasonable onset window, we define the onset window start time as the mean exponential growth start time (the mean of the individual wave numbers displayed in Figure 2c) for all *k*_lon_  ± 1.5*σ*, where *σ* is the standard deviation of the growth start times over all *k*_lon_. This criteria ensures that wave numbers which start to grow much earlier or much later than substorm onset are not taken into account, as we assume they are not part of the linear evolution of the instability. The linear stage of an instability is when the wave amplitudes grow exponentially in time [*Treumann and Baumjohann*, 1997]. The duration for which each individual wave mode exhibits exponential growth as found by the linear fitting algorithm is shown by the colored bars in Figure 2d. The colored bars represent the growth rate that each mode has. The onset window start time is denoted by the first vertical black line (average start time over all *k*_lon_ as discussed above), and the second vertical black line denotes the time at which the auroral beads expand poleward outside the analysis field of view marked in white in Figure 1. Finally, Figure 2e shows growth rates as a function of *k*_lon_ in the ionosphere (*k*_lon,*i*_) and the magnetosphere (*k*_lon,*m*_). From this plot we can infer the most unstable wave number, the wave number which exhibits the highest growth rate. This wave number and corresponding growth rate allows us to compare with plasma instability theory (see section 3) in order to identify which instability agrees with our observations of the highest growth rates at specific spatial scales.

Figure 2 demonstrates that although the sudden brightening of the auroral arc can be visually identified at 04:57:30 UT, the analysis of the spectral content of the aurora shows that exponential growth of individual wave numbers commences around 04:56:15 UT. The growth rates peak at 0.045 s^−1^ at longitudinal wave numbers measured in the ionosphere of *k*_lon,*i*_=2.0 × 10^−4^ m^−1^ in this event, or *k*_lon,*m*_=6.0 × 10^−6^  m^−1^ when mapped into the magnetosphere using a T96 model [*Tsyganenko*, 1995].

## 3 Statistics of Auroral Beads

We use the technique outlined in the previous section to analyze the growth rates and spatial scales of each of the 17 isolated substorm and pseudo‐breakup onset arcs that contained visually identifiable auroral beads which form along a preexisting arc. We note that the auroral beads in our identified events always form along a preexisting arc, which brightens and corresponds to the substorm onset arc. Hence, beading, preexisting arc, and substorm onset arc all refer to the same arc. We limit these events to those whose longitudes are close to the center of the field of view of the ASIs so that the beads are generated within the analysis box and remain in the same ASI for the duration of the exponentially growing phase. Table 1 provides our event list and relevant characteristics including magnetic local time (MLT), magnetic latitude and longitude of the arc, and direction of bead propagation. These characteristics were all identified from the auroral data only. Of particular note is that all 17 wave‐like auroral events occurred in the premidnight sector. There is no consistent azimuthal phase propagation; the direction of bead propagation varies between eastward (eight events), westward (three events), both directions (three events), and nonpropagating (three events), and so there is only a slight preference toward Eastward propagation (i.e., toward midnight in the premidnight sector). The magnitude of growth rates measured varies widely between events; maximum growth rates range over an order of magnitude between 0.03 and 0.3 s^−1^, with a median growth rate of 0.05 s^−1^. However, for each individual event it was usually possible to discern a peak in growth rates at a particular spatial scale. The upper growth rates are not limited by the frequency of the ASI as we require a minimum duration of growth of 30 s. This allows us to observe growth rates above the cadence of our imager.

**Table 1:** Event List—The Substorm and Pseudo‐Breakup Event List Used in This Study^a^

Date	ASI Station	Time (UT)	MLT	Arc MLAT	Arc MLON	Bead Propagation
28 Mar 2008	GILL	05:36:00	22:26:00	66.2–66.8	−33.0 to −22.0	Eastward
28 Nov 2005	FYKN	10:08:00	22:56:00	64.5–66.0	−100.0 to −90.0	Eastward
27 Jan 2006	FYKN	10:00:00	22:52:00	66.0–67.4	−100.5 to −91.5	None
22 Feb 2006	FSMI	06:26:30	21:32:00	66.4–67.1	−60.0 to −52.0	Westward
28 Feb 2006	WHIT	09:09:30	22:40:00	66.5–67.2	−88.0 to −80.0	Eastward
14 Feb 2007	GILL	05:07:00	22:24:00	64.9–65.8	−35.0 to −20.9	Eastward
7 Mar 2007	SNKQ	05:50:00	23:35:00	64.9–66.1	−15.0 to −5.5	Eastward
2 Oct 2008	SNKQ	04:29:00	22:56:00	66.8–67.15	−8.0 to −2.0	None
3 Jan 2009	GILL	04:36:00	21:18:00	66.7–67.2	−35.0 to −24.0	Westward
24 Feb 2009	FSIM	07:32:00	21:50:00	67.3–67.6	−70.0 to −63.0	None
15 Mar 2009	GILL	04:28:00	21:36:00	67.7–68.2	−30.0 to −20.0	Westward
7 Mar 2010	GILL	05:15:00	22:08:00	64.8 ‐ 66.0	−39.0 to −25.0	Both
31 Dec 2010	FSMI	06:37:00	21:22:00	66.2 ‐ 67.1	−64.0 to −53.0	Eastward
8 Mar 2011	GILL	06:24:00	23:06:00	66.9 ‐ 67.3	−38.0 to −27.0	Eastward
2 Oct 2011	GILL	04:55:00	21:16:00	67.8 ‐ 68.4	−45.0 to −15.0	Eastward
23 Mar 2008	GILL	05:44:00	22:24:00	67.4 ‐ 68.0	−31.0 to −25.0	Eastward
26 Feb 2008	RANK	04:50:00	21:22:00	69.3 ‐ 71.0	−35.0 to −22.0	Both

a Includes date, ASI station, substorm time and MLT, onset arc initial magnetic latitude and longitude, bead propagation direction, and whether this auroral arc brightened but did not expand poleward (pseudo‐breakup) or whether the arc expands poleward and “breaks up” (substorm).

Using global auroral imaging, *Henderson* [2009] estimated the growth rate of 0.005 s^−1^ from the total auroral intensity changes over three consecutive images spanning 4 min. *Henderson* [2009] notes that *as described by* *Cowley and Artun* [1997], *the growth could have been associated with an even faster “explosive” instability that leads to a “detonation”*. Since our ASI analysis is at a significantly higher temporal resolution and we can resolve individual wave numbers, we conclude that it is very likely that *Henderson* [2009] has indeed underestimated the growth rates. We discuss the ramifications of this result further below.

**Figure 4:**
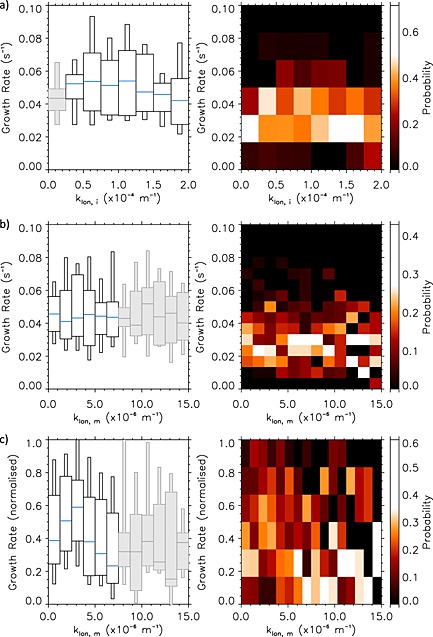
(left) A boxplot statistical analysis of growth rate as a function of spatial scale, where medians are denoted by the blue line, the large boxes represent the range of upper and lower quartiles, and the smaller boxes represent the upper and lower deciles and (right) growth rate probability occurrence plot as a function of (a) wave number *k*_lon,*i*_ measured in the ionosphere, (b) *k*_lon,*i*_ mapped to space using T96 magnetic field model, *k*_lon,*m*_, and (c) growth rates normalized to maximum growth rate for each event as a function of *k*_lon,*m*_. Subscripts *i* and *m* denote ionosphere and magnetosphere, respectively. Note that in order to render meaningful statistics, we group spatial scales into larger bins than are observed in (Figure 4a) and (Figure 4b). The boxes shown in grey indicate that less than 20 points are represented in this wave number range.

Figure 4 shows growth rates as a function of *k*_lon_ in two formats. Figure 4 (left) shows box plots of the statistical analysis of growth rate as a function of spatial scale, where median occurrence is highlighted as blue horizontal lines, the large boxes represent the range of upper and lower quartiles (25th–75th percentiles) and the smaller boxes represent the upper and lower deciles (10th–90th percentiles). Figure 4 (right) shows the probability occurrence statistics of growth rate as a function of spatial scale to demonstrate how likely a particular growth rate and *k*_lon_ will be observed.

Figure 4a shows statistics of growth rates as a function of ionospheric wave number, *k*_lon,*i*_, which are calculated assuming an emission height of 110 km altitude. It is evident from both the (left) median and (right) probability distributions that growth rates as a function of ionospheric wave number appear relatively flat and the median varies between 0.04 and 0.05 s^−1^ as a function of *k*_lon,*i*_. The Mann‐Whitney U test confirmed that the small difference observed in median growth rates is not statistically significant [*Mann and Whitney*, 1947]. This means that there is no preferred or more unstable wave number than others as deduced solely from ionospheric measurements.

We propose that auroral beads are the ionospheric manifestation of a magnetospheric plasma instability, as previously concluded by *Rae et al.* [2010] and *Motoba et al.* [2012]. To investigate the growth and structuring of magnetospheric waves that could be responsible for these ionospheric auroral beads, we map the azimuthal bead structure from the ionosphere into the equatorial plane of the magnetosphere. We use the Tsyganenko 1996 (T96) magnetic field model which depends upon solar wind dynamic pressure and *y* and *z* components of the interplanetary magnetic field and the geomagnetic Disturbance Storm‐Time index (*Dst*) [*Tsyganenko*, 1995]. Magnetospheric mapping during highly dynamic substorm times is unreliable; however, magnetospheric mapping is important in this study in order to estimate the magnetospheric wave number and remove latitudinal effects from the scaling of the ionospheric wave number. Equilibrium magnetic field mapping cannot be assumed to be reliable at substorm times due to the stretching of the tail as flux builds up in the lobes during the substorm growth phase. This means that field line stretching is likely to be underestimated. We chose only events that demonstrate steady equatorward motion of the growth phase arc prior to rapid auroral brightening, indicative of a classic substorm growth phase [*McPherron*, 1970]. This will not eliminate errors; however, this allows us to assume that the magnetic field model systematically underestimates substorm auroral bead spatial scales in the magnetosphere. The mapped spatial scales are therefore directly comparable between events even if the absolute value is likely to be lower than its actual magnitude [*Pulkkinen et al.*, 1991]. Using the T96 model to estimate the source location of the auroral arcs, we find that the arcs map to a range of distances between 8 and 18 *R*_*E*_ in the equatorial plane of the magnetosphere, with the majority lying between 9 and 12 *R*_*E*_. Beyond 9 *R*_*E*_ the model predicts magnetic field strengths in the plasma sheet which are <20 nT.

Using this assumption, Figure 4b shows the statistics of mapping *k*_lon,*i*_ along a T96 magnetic field to estimate *k*_lon,*m*_. Again, growth rates appear relatively flat as a function of azimuthal wave number, suggesting that there is no preferred wave number observed during these events in the magnetosphere either. This might be a result of the tail being in differing states during each substorm creating a continuum of unstable wave numbers; statistically, this would result in the flat distribution we observe. However, the Mann‐Whitney U test on this distribution suggests that the growth rates in the ranges *k*_lon,*m*_= 2.5–5.0 × 10^−6^ m^−1^ are larger than the others and that this result is statistically significant to a 95% certainty.

As noted previously, in general, there is a well‐defined peak in growth rate in individual case studies, but the size of the growth rate varies dramatically from event to event, by an order of magnitude. Assuming that a specific magnetospheric instability explains the azimuthal auroral beading and auroral substorm onset, it is entirely conceivable that the rate of growth is dependent upon unknown magnetospheric parameters such as plasma density or temperature [*Forsyth et al.*, 2014] or that solar wind driving affects the ionospheric response [*Sergeev et al.*, 2014]. In other words, even though we cannot determine the specific magnetotail characteristics during each substorm, we assume that a single magnetotail instability could explain our results and investigate the implications. It must be noted that our observations demonstrate that only one instability is operating in the first few minutes of auroral beading since the exponential growth of each k‐mode exhibits only one well‐defined growth rate during this interval. After the aurora expands outside of our analysis domain, any number of additional instabilities may be operating.

Hence, in Figure 4c we normalize the growth rates during each event to the largest growth rate in that event to investigate whether the magnetospheric spatial scales are repeatable across events. By assuming that the same instability can grow at different rates, Figure 4c shows a discernible peak in growth rates at *k*_lon,*m*_≈ 2.5–3.75 × 10^−6^ m^−1^ in both occurrence and medians, which corresponds to an azimuthal magnetospheric wavelength of *λ*_⊥_≈ 1700–2500 km (where *λ*_⊥_=2*π*/*k*_lon,*m*_). This is comparable to the ion gyroradius in a 6–9 nT field and therefore provides evidence that the ions may play an important part in the evolution of the instability. The Mann‐Whitney U test confirms that the peak observed in this wave number range is statistically significant to a 98% certainty when the growth rates are normalized. We reiterate that the wavelength is likely to be underestimated due to magnetospheric mapping during the substorm growth phase, discussed above [*Pulkkinen et al.*, 1991]. We note that using a different empirical magnetic field model such as T89 does not change the result that there is a distinct peak of growth rates with magnetospheric wave number across a similar range.

## 4 Comparison With Candidate Plasma Instabilities

Previous studies of auroral beads suggest that this ionospheric phenomenon is triggered by a magnetospheric instability. However, there has been no explicit quantitative and statistical comparison of values of the temporal (i.e., growth rates) and spatial (i.e., azimuthal wave numbers) evolution of the beads in order to compare with instability theory.

*Lui*, [2004, and references therein] identified numerous plasma instabilities which may be involved in the initiation of substorm onset. Our observations allow us to rule out several promising plasma instabilities for our substorm events: (1) The tearing instability [*Coppi et al.*, 1966] and the drift kink/sausage instability [*Zhu and Winglee*, 1996] have too slow growth rates and a radial *k* structuring; (2) the current‐driven Alfvénic instability [*Perraut et al.*, 2000] and lower hybrid drift instability [*Yoon et al.*, 1994] predict growth rates and frequencies which are larger by an order of magnitude than those observed. However, in a previous study of an isolated event, *Rae et al.* [2010] were unable to rule out the Kelvin‐Helmholtz instability which is predicted to have growth rates that peak at low *k*_lon_ by *Yoon et al.* [1996]. Our statistical observations allow us to rule this out, because the growth rates associated with this instability are over an order of magnitude greater than the rates we observe [*Hallinan and Davis*, 1970]. These instabilities have been ruled out on a combination of the growth rate magnitude and the spatial structuring of the excited waves. This means that the systematic errors acquired by magnetospheric mapping do not affect this conclusion.

This leaves the Cross‐Field Current Instability [*Lui et al.*, 1991; *Lui*, 1996, 2004] and the Ballooning Instability [*Voronkov et al.*, 1997; *Pu et al.*, 1999; *Zhu et al.*, 2004], both of which can explain azimuthal structuring of the onset arc and growth rates consistent with time scales observed. We directly compare Shear Flow Ballooning Instability [*Voronkov et al.*, 1997] and Cross‐Field Current Instability with our observations.

The challenge with studying the plasma instabilities invoked in substorm onset is to determine where the instability is initiated in the magnetotail. The Cross‐Field Current Instability as outlined in *Lui et al.* [1991] is studied using plasma sheet parameters observed in a statistical study of 15 current disruption events outlined in *Lui et al.* [1992] at radial distances of 7.4–8.8 R_*E*_. As previously stated we estimate that the auroral onset arcs do not map this close to Earth, but to the region 9–12 R_*E*_ typically associated with the substorm onset initiation. This location is where the field changes from dipole‐like to a more stretched tail‐like configuration [*Samson et al.*, 1992a; *Rae et al.*, 2014]. Hence, the current disruption events observed from space in *Lui et al.* [1992] may have been initiated at larger radial distances in the tail than inferred. Later, the instability is observed closer to Earth as the substorm current wedge (SCW) expands radially and azimuthally. *Lui et al.* [1991] present growth rates as a function of magnetospheric wave number of the Cross‐Field Current Instability in the near‐Earth and midtail plasma sheet. In the near‐Earth region the B_*z*_ component of the magnetic field is 25 nT. Assuming a T96 field; *B*_*z*_=25 nT maps to ∼8.5 *R*_*E*_ in the tail. This agrees with the locations where the instability was observed by *Lui et al.* [1992]. Hence, the substorm onset arc and location of the auroral beading is broadly consistent with the magnetic field magnitudes in the transition region between stretched and dipolar field lines [*Samson et al.*, 1992a]; *Lui* [1991], although ∼8.5 *R*_*E*_ is closer than our field mapping implies. In the midtail region *Lui et al.* [1991] selects 5 nT for the B_*z*_ component of the magnetic field, which corresponds to ∼13 *R*_*E*_ in the tail using T96. There is a similar problem with the Shear Flow Ballooning Instability as described by *Voronkov et al.* [1997], which does not quantitatively specify a region where the instability is likely to be triggered, but simply states *the inner edge of the plasma sheet* where *magnetic field lines are slightly stretched tail ward*. The analysis of *Voronkov et al.* [1997] uses B_*z*_ = 40 nT which, from the T96 model maps to 7.6 *R*_*E*_ downtail. However *Zhu et al.* [2004] find that the Ballooning Instability is excited for plasma *β* values in the range of ∼1–100. In plasmas with a higher *β* the high plasma pressure and therefore compression stabilizes the linear Ballooning Instability. The plasma parameters given by *Lui et al.* [1991, 1992] give a beta value of *β* = 4.4 which lies in this range. However, it is unclear how different magnetic field strengths affect the growth rates of this instability. There is a large region of the plasma sheet that satisfies these *β* values [*Walsh et al.*, 2013], which suggests that a large area of the plasma sheet could be unstable to the Ballooning Instability. In order to investigate whether it is possible for this instability to be triggered with lower B_*z*_ a full analysis of the relevant equations is required, which is beyond the scope of this work and will be explored in future.

### 4.1 Cross‐Field Current Instability

The Cross‐Field Current Instability (CFCI), as its name suggests, obtains its free energy from the cross‐field current due to an increase in resistivity in the near‐Earth region of the inner plasma sheet when the edge of the plasma sheet moves earthward during the substorm growth phase. The plasma sheet thins down to a thickness comparable with an ion gyroradius, allowing the ions to become demagnetized and drift duskward while electrons remain frozen to magnetic field lines. The instability takes the form of an Ion Weibel Instability (IWI) [*Lui et al.*, 1993] with wave numbers parallel to the background magnetic field and the Modified Two‐Stream Instability (MTSI) with wave numbers perpendicular to the background magnetic field *Lui et al.* [1991]. The angle of the waves excited is dependent on the relative ion drift speed. Higher *θ* (more perpendicular) waves are generated at lower drift velocities (*V*_0_), corresponding to the domination of the MTSI. The more parallel propagating waves (IWI) excited at higher drift velocities have shorter wave numbers (*k*). If the IWI mode is suppressed by a thin current sheet, then the MTSI will dominate leading to a more perpendicular wave propagation [*Lui et al.*, 1991]

**Figure 5:**
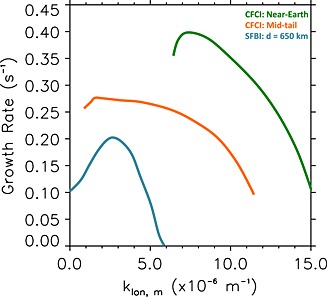
The growth rates as a function of wave number for the Cross‐Field Current Instability with inner‐edge (green) plasma sheet parameters: *V*_0_=0.5*v*_*i*_, *T*_*e*_=3  keV, *T*_*i*_=12 keV, and *n*_*e*_=*n*_*i*_=0.6 cm^−3^, and midtail (orange) plasma sheet parameters: *V*_0_=*v*_*i*_, *T*_*e*_=0.2 keV, *T*_*i*_=2 keV, and *n*_*e*_=*n*_*i*_=0.3 cm^−3^. The growth rates as a function of wave number for the Shear Flow Ballooning Instability (blue), where *ρ* = 4.06 × 10^−21^ kg m^−3^, *B* = 40 nT, and shear flow width, *d* = 650 km. The SFBI predicts lower growth rates than the CBCI with a peak at wave numbers of *k*_lon,*m*_≈3.0 × 10^−6^ m^−1^.

*Lui et al.* [1991, 1992] investigate the CFCI using parameters representative of the inner‐edge and midtail region of the plasma sheet. For the inner‐edge *V*_0_=0.5*v*_*i*_, *n*_*e*_=*n*_*i*_=0.6 cm^−3^, *T*_*i*_/*T*_*e*_=4*T*_*i*_=12 keV, and *B*_*z*_=25 nT. For the midtail region *V*_0_=*v*_*i*_, *n*_*e*_=*n*_*i*_=0.3 cm^−3^, *T*_*i*_/*T*_*e*_=10*T*_*i*_=2 keV, and *B*_*z*_=5 nT. Note that a full analysis of all parameters is beyond the scope of this work and will be explored in future with added constraints from spacecraft data. Figure 5 shows the growth rates as a function of wave number from both the inner‐edge and midtail plasma parameters. The growth rates for the inner‐edge parameters are higher in comparison to our auroral observations. However a clear peak in growth rates can be observed at *k*_lon_=7.0 × 10^−6^ m^−1^. The maximum growth rate for the midtail parameters is lower; however, the growth rate distribution is almost flat at low wave numbers. *Lui et al.* [1991] calculate the maximum growth rates for a variety of drift velocities. These are shown in Table 2 and demonstrate that the growth rates predicted in the near‐Earth plasma sheet are much too high. The maximum rate for the midtail plasma sheet with a drift velocity of *V*_0_=0.3*v*_*i*_ is more consistent with our observations.

**Table 2:** Table of Maximum Growth Rates Predicted for Different Drift Velocities for Waves in the Near‐Earth and Midtail Current Sheet From *Lui et al.* [1991]

*V*_0_/*v*_*i*_	0.3	0.5	1.0	2.6	9.0
*γ*‐ midtail	0.052			0.62	2.0
*γ*‐ near‐Earth		0.36	1.12		

**Figure 6:**
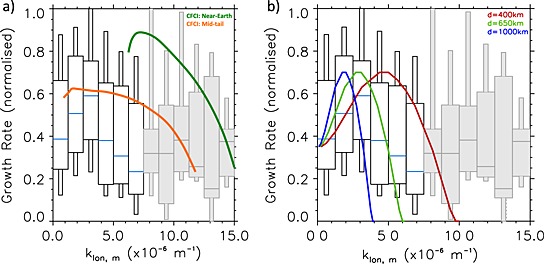
The normalized growth rate as a function of spatial scale for (left) the cross‐field current instability for inner‐edge plasma sheet parameters (green) where *V*_0_=0.5*v*_*i*_, *T*_*e*_=3  keV, *T*_*i*_=12 keV, and *n*_*e*_=*n*_*i*_=0.6 cm^−3^ and midtail plasma sheet parameters (orange) where *V*_0_=*v*_*i*_, *T*_*e*_=0.2  keV, *T*_*i*_=2 keV, and *n*_*e*_=*n*_*i*_=0.3 cm^−3^. (right) Shear Flow Ballooning Instability, where *ρ* = 4.06 × 10^−21^  kg m^−3^, *B* = 40 nT. Keeping these parameters constant, different growth rate curves are obtained by varying the width of the shear flow region. The growth rate curves have been normalized to 0.7 which corresponds to a growth rate of 0.2 s^−1^ to facilitate qualitative comparison with the normalized growth rates from observation. The boxes shown in grey indicate that less than 20 points are represented in this wave number range.

Figure 6a shows a comparison of our statistical results with the characteristics of the CFCI for varying plasma sheet locations. Our statistical results demonstrate maximum growth rates at small wave numbers. The magnitudes of the growth rates are in better agreement with the midtail parameters; however, the observed variation of growth rate with wave number is not replicated by the CFCI.

In summary, using plasma sheet parameters indicative of the midtail magnetotail region with low drift velocities, the CFCI predicts growth rate magnitudes of the same order as those inferred from auroral growth rates. At higher *B*_*z*_ corresponding to close to the inner edge of the plasma sheet, the peak in growth rate becomes more pronounced, but occurs at larger wave numbers and higher growth rates than inferred. The growth rates for the midtail parameters do not exhibit a clear peak in the growth rates we infer when assuming that the beads are the signature of the same instability. Further investigation of the effect of changing the parameters needs to be done in order to definitively rule this instability in or out.

### 4.2 Shear Flow Ballooning Instability

The Shear Flow Ballooning Instability (SFBI) is a hybrid instability incorporating the Kelvin‐Helmholtz instability, driven by small‐scale shear flows and the Rayleigh‐Taylor instability, driven by large‐scale earthward directed pressure gradients. Strong azimuthal shear velocities have been observed in the equatorial regions of field line resonances. For example, *Samson et al.* [1996] report of shears up to 200 km^−1^ over radial distances of the order of 0.1 *R*_*E*_. The hybrid SFBI possesses significantly faster growth rates and shorter time scale exponential growth than a pure Kelvin‐Helmholtz mode, making it a suitable candidate to compare with the growth rates obtained from our optical analysis. The substorm onset arc is tied to the boundary between stretched and more dipolar field at the inner edge of the plasma sheet [*Samson et al.*, 1992b] and in precisely the region where pressure gradients control the physics behind the Shear Flow Ballooning Instability.

The MHD equations for the radial component of the shear flow velocity *V*_*x*_ is given by

(1) $$V_{x}^{\prime \prime }=k^{2}V_{x}\left(1-\frac{V_{0}^{\prime \prime }}{k(\omega -kV_{0})}-\frac{W}{{\left(\omega -kV_{0}\right)}^{2}}\right)$$

where

$$W=-\frac{g\rho_{0}^{\prime }}{\rho_{0}}-\frac{g^{2}}{V_{f}^{2}}$$

and *ω* − *k* *V*_0_(*x*) is a Doppler‐shifted wave frequency, $V_{f}^{2}=C_{s}^{2}+V_{a}^{2}$ is the square of the fast mode velocity, *C*_*s*_ is the acoustic velocity, *V*_*a*_ is the Alfvén velocity, and *V*_0_(*x*) the shear flow velocity, $V_{x}^{\prime \prime }$ and $V_{0}^{\prime \prime }$ denotes the second derivative with respect to *x* and *g* is the centripetal acceleration of the particles as a result of magnetic curvature and particle inertia. When *W* > 0 the pressure gradient is stable, and for *W* < 0 it is unstable and hence able to take part in substorm onset.

Using magnetic field component: *B*_*z*_=40 nT and plasma sheet mass density *ρ* = 4.06 × 10^−21^ kg m^−3^ as given in *Voronkov et al.* [1997], we find that the growth rate peaks at 0.2 s^−1^ and there is an inverse relationship between the most unstable spatial scales and the size of the shear flow region. This is in contrast to the CFCI, where an increase in magnetic field strength or ion drift velocity increases the wave number at which the growth rate peaks. This is shown in Figure 5 where the absolute growth rates predicted by the SFBI and CFCI are compared. The growth rates as a function of wave number for the CFCI presented in *Lui et al.* [1991] with inner‐edge and midtail plasma sheet parameters are shown in comparison to the growth rates to the SFBI growth rates from *Voronkov et al.* [1997] for a shear flow width of *d* = 650 km.

Figure 6b shows a comparison of our statistical results with the characteristics of the SFBI for varying shear flow regions. Our statistical results demonstrate maximum growth rates at small spatial scales which agree well if the SFBI was driven by a shear flow width in the magnetosphere of 600–700 km. This is an extremely localized region in the magnetosphere, but we should note that if the spatial scales of the instability have been underestimated due to the errors in magnetospheric mapping, this would also underestimate the size of the shear flow region predicted.

Our analysis of the SFBI suggests that some combinations of plasma and magnetic field characteristics are able to explain our observed results. This indicates that the SFBI could be the cause of the substorm onset arc.

## 5 Discussion and Conclusion

The optical analysis technique presented in this paper provides a quantitative method to remote sense the physics of substorm onset from spatial analysis of substorm‐related aurora. In the ionosphere, we have observed the auroral beads with wavelengths of ∼60 km, evolving to ∼120 km, in agreement with previous individual case studies, e.g., *Friedrich et al.* [2001], *Sakaguchi et al.* [2009], and *Rae et al.* [2010]. The statistical analysis of multiple auroral brightenings has yielded vital new constraints on the nature of the plasma instability associated with substorm onsets and pseudo‐breakups.

Specifically, we find that

1. The statistical result of the analysis of auroral spatial scales demonstrates the most unstable azimuthal wavelength of the magnetospheric instability is at least *λ*_⊥_≈1700 − 2500 km;
2. The most unstable spatial scales have growth rates ranging from 0.03 to 0.3 s^−1^ with a median growth rate of 0.05 s^−1^;
3. The Cross‐Field Current Instability in the near‐Earth plasma sheet predicts growth rates which are too high and at much smaller azimuthal scales (or larger k) to explain our observations;
4. The Cross‐Field Current Instability in the midtail region (*B*∼5 nT) with a drift velocity *V*_0_=*v*_*i*_ agrees better with the magnitude of the inferred growth rates; however, the theoretical growth rates at the same magnetic field strength do not show a clear peak at the right wave number as observed. Lower drift velocities (*V*_0_=0.3*v*_*i*_) predict growth rates closer to those observed;
5. The Shear Flow Ballooning Instability with a localized shear flow region of ∼650 km and plasma sheet magnetic field strength of 40 nT can explain our observed results.

More work is necessary to fully investigate the range of plasma and magnetic field conditions that may support the instabilities identified by our analysis of the substorm aurora.

Even though the CFCI predicts waves at similar temporal and spatial scales, further analysis of the plasma characteristics is required in order to conclude whether combinations of the plasma sheet parameters and drift velocities can predict a peak in growth rates at the spatial scales we observe.

In our analysis we assumed that the same instability was acting in the magnetotail for each event. This would result in the same shape of growth rate as a function of wave number, although the magnitude of growth may be different in each instance. Assuming that only one instability is causing the substorm onset arc suggests that the instability most likely to play a part in the trigger of substorm onset is the Shear Flow Ballooning Instability, as the peak growth rate of 0.2 s^−1^ at spatial scales of *k*_lon_=2.5 − 3.75 × 10^−6^ m^−1^ is predicted by this instability with a shear flow region of ∼650 km. The effect of different plasma parameters such as density, *B*_*z*_, and pressure gradient on the growth rate amplitude and shape as a function of wave number requires further investigation. However, if this assumption is incorrect and the instabilities occurring in each event are different, then this normalization is unjustified. Without any additional information on the magnetotail plasma and magnetic field state, we cannot explore whether only one instability could be responsible for generating auroral beads.

The purpose of this manuscript is to statistically show that the formation and evolution of auroral beads are a signature of the linear stage of an instability. We have used our analysis to provide the characteristics of the growth rates and spatial scales of the most unstable wave numbers of this instability. However, how the instability accelerates auroral electrons to form the auroral beads we observe is the next logical step.

We show for the first time a quantitative comparison between observations of the spatial and temporal structuring of the substorm onset arc and its relation to proposed magnetotail instability mechanisms. We statistically demonstrate the evolution in space and time of the substorm onset arc, providing the clearest indication yet that the substorm onset arc itself is both wave driven and is inextricably linked to a magnetotail instability. The auroral beads exhibit exponential growth across a broad range of spatial scales in the ionosphere initially suggesting that there are no preferential spatial scales for auroral bead growth. However, when we make two relatively simple and reasonable assumptions, that magnetic field mapping introduces a systematic error and that substorms can grow at different temporal rates, we find that there is indeed a preferred *k* spectrum peaking at low wave numbers. To provide further evidence that we are measuring the ionospheric optical manifestation of a magnetospheric instability in situ space measurements are required. Our results provide the strongest evidence yet that the substorm onset arc is created by a plasma instability such as the Shear Flow Ballooning Instability [*Voronkov et al.*, 1997]. We use a combination of ground‐based data and magnetic field mapping to predict the location of the instability in space and its spatial scales. By doing so, we provide important estimates of the characteristics of the magnetotail region driven unstable during the substorm and containing the substorm onset arc. Using these predictions, we suggest the first observational test in the magnetotail that could finally identify the magnetospheric source of the substorm plasma instability and ultimately the cause of the substorm onset arc itself.

## Supporting information

Movie S1

Text S1
