# Supplementary material for: Statistical characterization of the growth and spatial scales of the substorm onset arc
Source: J Geophys Res Space Phys. 2015 Oct 20;120(10):8503–16. doi: 10.1002/2015JA021470 (PMC5111420; doi:10.1002/2015JA021470)
Supplement: Supplementary file 2 — Text S1 [file JGRA-120-8503-s002.pdf]

# Supporting Information for "Statistical Characterisation of the growth and spatial scales of the substorm onset arc"

N.M.E. Kalmoni[1], I.J. Rae[1], C.E.J. Watt[2], K. R. Murphy[3], C. Forsyth[1], C. J. Owen[1]

**Contents of this file**

1. Movie S1

**Introduction**

**Movie S1.** Movie of substorm event at GILL on 2011-10-02.

---

[1]Mullard Space Science Laboratory, University College London, Holmbury St. Mary, Dorking, RH5 6NT, UK

[2]Department of Meteorology, University of Reading, Reading, UK

[3]NASA Goddard Space Flight Center, Greenbelt, ML, USA
